# The Execution of a Woman

**Published:** 1899-05

**Authors:** 


### THE EXECUTION OF A WOMAN.

AT SING SING prison on March 20, 1899, there was legally killed a woman named Martha Place, who had brutally and fiendishly murdered her step-daughter and attempted to murder her husband. She was electrocuted after vain efforts had been made to secure a commutation of her sentence at the hands of Governor Roosevelt, who, however, with that firm determination which has always characterised him, refused to interfere. There was no sex in crime in this case. There was a question regarding Mrs. Place's sanity at one time, but Governor Roosevelt caused her to be examined by Drs. Dana and Polk and they reported that she was sane. The electrocution was successful in every respect. There were no harrowing scenes and death was instantaneous.

The last woman legally killed in this state was Mrs. Druse, at Herkimer, who was hanged and who fought her keepers every inch of the way from the cell to the gallows, creating a scene of horror which has ever since lived in the memory of the witnesses.

Electrocution is now established as the most humane method of execution and the idea was born in Buffalo, in the brain of the late Dr. A. P. Southwick. New York State first adopted it, then Ohio took it up and now Massachusetts has done away with hanging and made electrocution the method of execution. When the question was befoie the Ohio legislature the fight was bitter. A newspaper published at Columbus, fighting for electrocution, sent to New York State for reports and secured from Dr. Nelson W. Wilson, of this city, a lengthy opinion. Dr. Wilson has for years made legal executions a study. He had seen a great many persons hanged and a number go to their death in the chair, so that he was qualified to speak more properly from the view point of an expert than any one else who had been approached. His paper pronounced in favor of the chair and one night it was read before the committee. The committee reported in favor of the new law the next day.

Other states will follow New York, Ohio and Massachusetts in establishing electricity as the method of execution and then there will be an end to the strangling and choking of murderers on the gallows.

